# Use of POCUS in Chest Pain and Dyspnea in Emergency Department: What Role Could It Have?

**DOI:** 10.3390/diagnostics12071620

**Published:** 2022-07-03

**Authors:** Andrea Piccioni, Laura Franza, Federico Rosa, Federica Manca, Giulia Pignataro, Lucia Salvatore, Benedetta Simeoni, Marcello Candelli, Marcello Covino, Francesco Franceschi

**Affiliations:** 1 Department of Emergency Medicine, Fondazione Policlinico Universitario A. Gemelli IRCCS, 00168 Rome, Italy; giulia.pignataro@gmail.com (G.P.); luciasalv@gmail.com (L.S.); benedetta.simeoni@policlinicogemelli.it (B.S.); marcello.candelli@policlinicogemelli.it (M.C.); covinom@gmail.com (M.C.); francescofranceschi@gmail.com (F.F.); 2 Facoltà di Medicina e Chirurgia, Scuola di Specializzazione in Medicina d’Emergenza-Urgenza, Università Cattolica del Sacro Cuore, 00168 Rome, Italy; cliodnaghfranza@gmail.com (L.F.); federosa1991@gmail.com (F.R.); fedemanca@gmail.com (F.M.)

**Keywords:** POCUS, point of care ultrasound, emergency medicine, ultrasound, emergency department, critical care, COVID-19, lung ultrasound, chest pain, dyspnea

## Abstract

Chest pain and dyspnea are common symptoms in patients presenting to the emergency room (ER); oftentimes it is not possible to clearly identify the underlying cause, which may cause the patient to have to return to the ER. In other cases, while it is possible to identify the underlying cause, it is necessary to perform a large number of tests before being able to make a diagnosis. Over the last twenty years, emergency medicine physicians have had the possibility of using ultrasound to help them make and rule out diagnoses. Specific ultrasound tests have been designed to evaluate patients presenting with specific symptoms to ensure a fast, yet complete, evaluation. In this paper, we examine the role of ultrasound in helping physicians understand the etiology behind chest pain and dyspnea. We analyze the different diseases and disorders which may cause chest pain and dyspnea as symptoms and discuss the corresponding ultrasound findings.

## 1. POCUS in Chest Pain: What Role Could It Have?

Over the last twenty years, ultrasound has gained growing importance in the emergency department. Initially, ultrasounds were usually performed on trauma patients to evaluate whether patients needed surgery when CT (computed tomography) scans were not as widely spread as they are now [1].

It quickly became obvious that emergency ultrasound, or point of care ultrasound (POCUS), has some very interesting aspects in the context of the emergency room (ER), allowing the physician to immediately obtain images of the patient during the first visit and thus rule out major pathologies immediately [2].

Ultrasound has gained popularity in many different conditions and clinical presentations, from critically ill patients [3] to patients with musculoskeletal pain [4].

While on the one hand ultrasound presents a number of advantages, it also presents some potential pitfalls, particularly because it is highly influenced by the operator’s ability to perform it [5].

However, particularly in overcrowded ERs, ultrasound can aid the emergency physician in the diagnosis and stratification of patients.

Chest pain is one of the main causes of admission to the emergency room, and it has been estimated that about 1 million people refer to the ER for this reason in Italy every year. It has been estimated that in about half of the cases the cause is cardiac, yet in the other half it is not [6]. While acute coronary syndrome (ACS) is the first cause that needs to be ruled out, there are a number of other conditions that can present with chest pain [7]. Additionally, the characterization of the pain, which should in theory aid the staff towards the correct diagnosis, is not reliable in a large number of patients due to a number of psychosocial and physiological factors [6].

Patients presenting to the ER with a complaint of chest pain need to be thoroughly examined and undergo a number of tests, studying not only cardiac function, but also ruling out abdominal or respiratory causes, particularly in those patients who cannot be safely discharged without a diagnosis [8]. Thus, diagnostic workup can take hours if not days.

While using ultrasound during the first visit may not allow the physician to immediately rule out acute coronary syndrome, it could identify other possible causes of the symptom, ranging from respiratory to cardiovascular to abdominal disorders.

In the present review, we discuss the possible echography pictures related to different pathologies which can present with chest pain and discuss possible applications.

## 2. Cardiac Causes

As discussed above, chest pain is the second most frequent cause of accessing the emergency room [7]. In case of suspicion of ischemic chest pain, the patient is evaluated with a 12-lead electrocardiogram and troponin dosage, which are necessary for the diagnosis of ACS [9]. In the event of major changes in the ECG (electrocardiogram) or troponin values, the patient is urgently evaluated by the cardiologist.

Once the diagnosis of ACS has been ruled out, there are many other cardiac clinical pictures in which the help of ultrasound can be decisive for the emergency room physician. There are, indeed, many other heart diseases that can occur in the emergency room with chest pain, such as aortic dissection, pericarditis, and pulmonary embolism.

The use of echocardiography by the emergency physician has several advantages, such as a targeted and early evaluation, and is rapid and non-invasive. However, this method, which has been spreading rapidly for several years, does not yet have a good degree of overall accuracy [10].

One of the possible differential diagnoses of cardiogenic chest pain is aortic dissection, for which CT angiography or trans-oesophageal echocardiography are the gold standards [11]. However, in some situations, such as severe renal insufficiency or failure to fast, these tests cannot be conducted, and a transthoracic echocardiogram (ETT) showing viewable aortic root dilation or an intimal flap can direct us towards the correct diagnosis [12]. The same signs are also present in the case of abdominal aortic dissection.

Unfortunately, a downside of ETT is that a negative echocardiography for aortic pathology does not rule it out completely [13].

**Figure 1:**
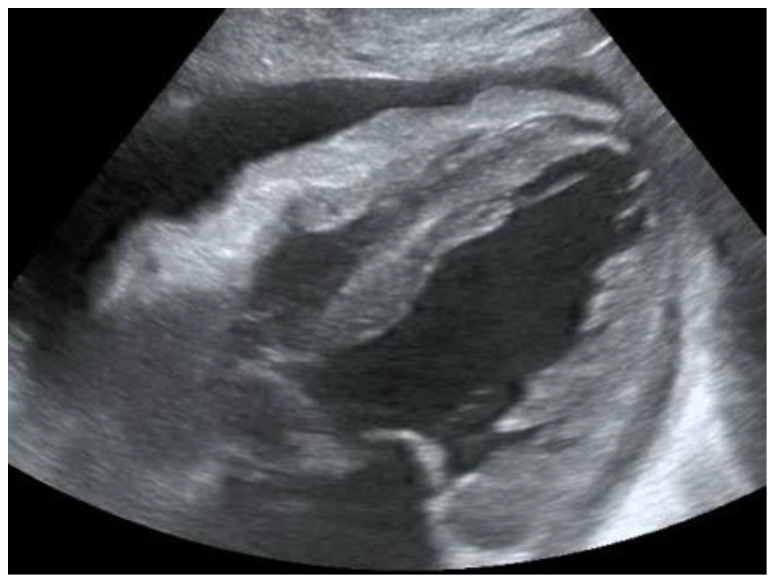
POCUS echocardiography: subcostal projection with pericardial effusion.

Another condition in which echocardiography can help the physician is the finding of hypoechoic material between the two layers of the pericardium, which allows us to make a diagnosis of pericardial effusion (Figure 1). Another advantage is that the ultrasound performed in the emergency room by the emergency doctor has a very high sensitivity (96%) and specificity (98%) towards this pathology [14].

Knowing how to identify a pericardial effusion also allows us to diagnose much more dangerous clinical pictures, such as cardiac tamponades [15].

Another condition characterized by chest pain which can often be similar to ischaemic pain is pulmonary embolism. We will further discuss the role of POCUS in the diagnosis of pulmonary embolism in the chapter on thoracic ultrasound.

**Figure 2:**
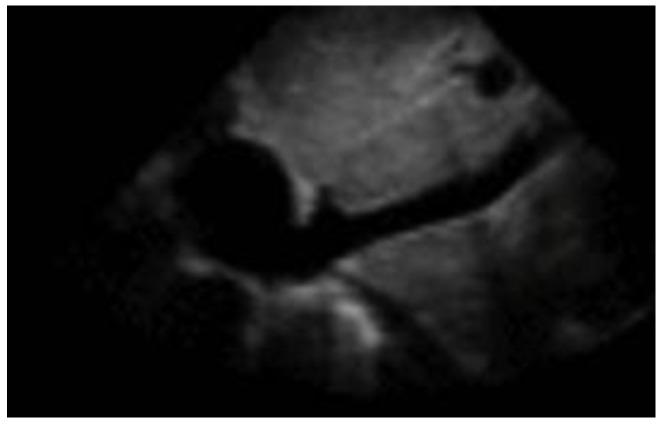
Subcostal longitudinal view: normal IVC.

While we will discuss heart failure further in the chapter on thoracic POCUS, it is worth noting that the evaluation of the IVC (Figure 2) can help us in several respects, as its diameter and collapsibility reflect the systemic volume state (Table 1) [16,17].

**Figure 3:**
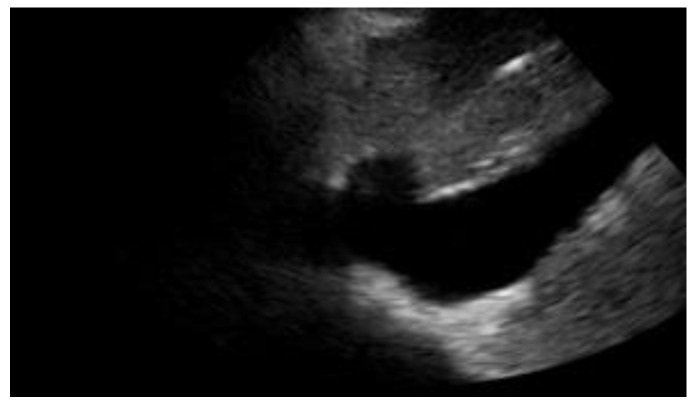
Subcostal longitudinal view: plethoric IVC. This finding suggests a state of fluid overload.

A reduction in diameter accompanied by an increase in collapsibility orients us, for instance, towards a state of hypovolemia, while an increase in caliber and a smaller excursion during inspiration indicates congestion. For this reason, finding a congested IVC may suggest fluid overload and lead to a diagnosis of heart failure [18] (Figure 3).

Additionally, IVC volume can help the clinician in evaluating the patient’s response to a fluid challenge [19].

Cardiac POCUS can also aid physicians who examine patients with chest pain who go into cardiac arrest [20]. Indeed, ACS is not the only possible underlying cause in this context, and properly trained emergency room doctors should be able to conduct an appropriate ultrasound examination in patients experiencing cardiac arrest [21].

In this situation, conducting an ultrasound examination is of fundamental importance for several reasons, in particular because we could identify some reversible causes of the arrest, such as the presence of cardiac tamponade or pulmonary embolism [22].

In our opinion, the use of POCUS (point-of-care ultrasound) by the emergency room physician therefore represents the future of emergency medicine.

In recent years, its use has progressively increased [23] and we have seen how fundamental it is in the evaluation of chest pain, in the differential diagnosis of cardiac pathologies, and in the correct management of cardiac arrest. 

For the proper use of POCUS, the emergency physician must also be aware of its limitations, such as being a highly operator-dependent examination which does not possess the same diagnostic accuracy as more advanced imaging techniques such as CT and MR.

The primary care physician should not make the mistake of overestimating his or her own capabilities, but should interpret POCUS as a tool that can help him or her in the initial framing of the patient, relying on more accurate imaging techniques and specialist consultation in more complex cases.

Overall, cardiac POCUS can help the physician in evaluating the presence of pericardial effusion, the overdistension of the right ventricle, the presence of aortic dissection, and also the degree of collapse of the IVC and the ejection fraction [24] (Table 2).

## 3. Chest Pain and Its Respiratory Causes

Chest pain often is associated with dyspnea or shortness of breath (SOB), which is in itself one of the most common causes of emergency room access. The origin of these symptoms is often sought in heart and lung diseases [25].

Patients presenting with these symptoms are routinely subjected to a chest X-ray examination, although in recent years we have witnessed the rapid growth and spread of thoracic POCUS, in which the physician himself, no longer the radiologist, performs the diagnostic examination.

One of the advantages includes knowing first-hand the clinical picture and, consequently, the symptoms reported by the patient. For this reason, it is easier to orientate towards the correct differential diagnosis [26].

It has been known for some time now that the application of this method also presents many advantages for the patient when it is applied for the study of the thorax, including a lower exposure to ionizing radiation, a greater speed of execution, and a reduction in cost [27].

One of the most well-established aspects of this approach is that integrating ultrasound into the normal diagnostic tests for patients who come to the emergency room for dyspnoea and chest pain improves diagnostic accuracy [28].

In this table, we collect some of the most common thoracic ultrasound signs in clinical practice (Table 3) [29].

There are numerous thoracic pathologies that can be identified with thoracic ultrasounds such as: pneumothorax, pleural effusion, pneumonia, pulmonary oedema and diaphragm dysfunction [30].

As discussed above, chest X-rays are routinely performed in the diagnostic evaluation of patients who come to the emergency room for chest pain and dyspnea, but numerous studies have shown that ultrasound is even superior to traditional radiology for the diagnosis of some clinical pictures [31,32,33,34].

With regard to pleural effusion, for instance, the greater diagnostic accuracy of thoracic ultrasound compared to X-rays in certain situations has been known for several years, both in terms of detecting it and quantifying it [33]. The most common method used in our emergency department to estimate the amount of pleural effusion present is to calculate 90 mL for each centimeter of cranio-caudal extension of the effusion with the probe oriented longitudinally in the dorso-lateral wall in the patient in the standing or sitting position (first Goecke formula) [35].

The accuracy of the ultrasound method for the diagnosis of pneumothorax and haemothorax is also supported by numerous pieces of evidence; many of these studies were conducted in patients who came to the emergency room for trauma and then underwent E-FAST (Extended Focused Assessment with Sonography for Trauma).

FAST ultrasound examination was initially designed to evaluate free intra-abdominal effusions in trauma patients and quickly spread in all trauma centers; it was then integrated with further scans to assess the presence of pneumothorax and haemothorax, becoming E-FAST [36]. Thus, ultrasound has had a significant impact on the diagnosis of post-traumatic complications such as pneumothorax and haemothorax. The possible use of thoracic ultrasound for the identification of injuries such as post-traumatic haemothorax and pneumothorax was also carefully analyzed in a meta-analysis, which established its accurate diagnostic validity [37]. As for the diagnosis of post-traumatic pneumothorax, this evidence was also confirmed in a recent meta-analysis which showed that thoracic ultrasound is even superior to traditional radiological examination [31]. The same conclusions were also reached in meta-analysis conducted in non-traumatized patients [32].

Thoracic POCUS has also gained importance in non-traumatic patients with a number of different conditions. A dangerous and prevalent condition in which the importance of POCUS is now consolidated with numerous pieces of evidence is the diagnosis of acute pulmonary oedema [38].

In a subject with symptoms suggestive of acute pulmonary oedema, the presence of B-lines at the ultrasound clearly points towards this diagnosis. A meta-analysis has confirmed the diagnostic superiority of ultrasound over traditional radiology for the diagnosis of acute pulmonary oedema [34].

We can therefore state that, in relation to these clinical pictures, namely, pneumothorax, pleural effusion, and pulmonary oedema, thoracic ultrasound has greater diagnostic accuracy, even higher than traditional radiology.

Another condition in which thoracic POCUS can significantly help in making a diagnosis is pulmonary embolism. Patients with this condition often present with chest pain and SOB, but presentation can vary widely [39].

Ultrasound findings supporting the diagnosis of pulmonary embolism include the findings of subpleural infarction, the dilation of the right heart cavities or the presence of thrombus within them, and the presence of deep vein thrombosis. While the ultrasound cannot lead us to the diagnosis of pulmonary thromboembolism, the gold standard of which is recognized as angio-CT [40], it can still be useful in properly stratifying patients to determine if they need to undergo CT angiography [41].

Ultrasound is also extremely useful in patients presenting with chest pain, cough, fever and dyspnea. These symptoms, associated with the presence of risk factors and an increase in inflammatory markers, can guide us towards the diagnosis of pneumonia, for which ultrasound has a high sensitivity, specificity and accuracy [42]. In particular, in very young or pregnant patients, ultrasound represents a valid option in diagnosing pneumonia [43].

Even the finding of a normal type A pattern without pleural or parenchymal changes can be suggestive of some pathologies, such as exacerbations of chronic obstructive pulmonary disease/asthma [43].

The evidence regarding the diagnostic accuracy of thoracic ultrasound in the ER has also been confirmed by the unexpected arrival of a new disease. Since December 2019, the SARS-CoV-2 virus has spread all over the world, presenting itself mainly in the form of interstitial pneumonia [44]. With the onset of the COVID-19 pandemic, the use of thoracic ultrasound has spread considerably [45].

Chest ultrasounds in emergency departments were immediate and valuable aids in the clinical management of interstitial pneumonia due to COVID-19 [46]. Ultrasound could be useful to isolate both patients with suspected acute respiratory failure due to COVID-19 pending the outcome of the nasopharyngeal swab and those with suspected infection with a suspected false-negative molecular swab [47].

As already mentioned for the generic thoracic ultrasound, the advantages over the use of CT are repeatability, the rapid execution of the examination, the absence of radiation, the cost and—in cases of very contagious viral disease—avoiding transporting the patient to the radiology rooms, considerably reducing the risk of infection for other healthcare professionals [48].

The most characteristic ultrasound findings found in COVID-19 pneumonia are the presence of diffuse B-lines and the irregularity of the pleural line [49].

The characteristics of clinical severity found in ultrasound can also be compared to those found in a much more accurate method, such as CT [50]. The validity of using thoracic ultrasound for the severity of infection has been confirmed in several studies, and also in recently published meta-analyses [51].

In this table, we summarize the ultrasound findings associated with the cited clinical patterns (Table 4).

In conclusion, the POCUS is a promising new tool in the possession of the emergency room physician to help in various differential diagnoses and, as we have discussed, it can prove to be of fundamental importance, especially in relation to particular clinical pictures (Table 5).

## 4. Abdominal Causes

Chest pain can sometimes be a manifestation of abdominal pathology. A common example of this is gastritis, which can present itself in a similar fashion to cardiac ischaemia, as epigastric tenderness or as pain resolution after the administration of proton pump inhibitors (PPIs). In association with a lack of risk factors, POCUS is often used to determine whether gastric disturbances can be the cause of chest pain. However, although uncommon, there have been cases of patients suffering from more severe diseases and conditions who were discharged with the diagnosis of gastritis, only to return shortly after to the attention of the emergency physicians [52,53].

While ultrasound is not typically used to diagnose this disorder, it can further support the diagnosis. In particular, the thickening of antral walls and mucosal layers can further support the diagnosis, even though the lack of these signs cannot safely exclude the diagnosis [54]. Overall, a sure diagnosis can only be made through endoscopy.

Another condition in which chest pain can be the main symptom is esophagitis. The most common cause is gastroesophageal reflux disease (GERD), but infections and autoimmune diseases can also determine this disease. Similarly to gastritis, endoscopy cannot be replaced by ultrasound in the diagnosis, nor can it rule out the diagnosis; however, the presence of a thickened oesophageal wall can support the diagnosis [55]. Another interesting finding is the presence of a slow, trickling reflux of gastric content after the patient swallows in those with GERD; however, once again endoscopy is the gold standard [56].

Ultrasound may not be conclusive for the diagnosis of inflammatory disorders of the oesophagus and the stomach, but it can offer significant help in other more severe disorders; in particular, oesophageal perforation can result in the non-visualization of the heart on an ultrasound due to the presence of air, and free fluid may also be present in the upper abdominal quadrants [57]. A similar presentation may also be present in the case of gastric rupture, in which the hyper echogenicity of the right anterior extrarenal tissue (renal rind sign) may also be present [58].

Other conditions involving abdominal organs can also first present with chest pain. Gallstones, for instance, can determine pain in the upper abdomen and chest, and POCUS in this case has up to 88% and 99% specificity [59]. In uncomplicated forms, the ultrasound is able to detect the stone and, if it is creating an obstruction, it is also possible to observe bile duct dilatation [60]. In the case of cholecystitis, ultrasound will show gallbladder distension, wall edema, and pericholecystic fluid collection, and is considered the gold standard for diagnosis [61].

Pancreatic disorders can also determine chest pain, usually in association with epigastric pain. While a number of conditions could potentially determine these symptoms, the pancreatic disorder of major concern for the emergency medicine physician is acute pancreatitis. An enlarged and oedematous pancreas is the pathognomonic finding, even though it is not easily seen, particularly during the first stages of inflammation [62]. Other findings that may be suggestive include peripancreatic fluid collections, venous thrombosis, arterial pseudoaneurysm, the presence of gallstones, or the dilatation of the biliary tree.

Additionally, kidney stones can sometimes present with chest pain, which is sometimes described as a constrictive pain, resembling the one of ACS. Ultrasound is the preferred imaging method, showing the stone itself or indirect signs of its presence, namely, ureter dilatation above the stone [63].

Overall, abdominal ultrasound can also provide some help to the emergency medicine physician evaluating chest pain. In Table 6, a short summary of the various causes that can be evaluated through abdominal ultrasound is presented.

## 5. Conclusions

Chest pain and dyspnea are common conditions in emergency settings, and it is often difficult to understand the underlying causes. Medical history, associated symptoms, laboratory tests and ECG are important in making a correct diagnosis, but in some cases they are not enough. Patients with pneumonia, gastritis, and pericarditis can all present with chest pain, which can sometimes be described in confusing ways for the physician [64].

While, in some cases, laboratory tests are then enough to understand the etiology of the symptom, in other conditions, tests can result negative or not determine any specific condition; thus, imaging has an important role in this context. Ultrasound, in particular, allows the emergency physician to immediately evaluate a number of conditions, to either rule them out or make a diagnosis (Table 7).

The use of ultrasound in this context should not be limited to the examination of the heart and lungs, but should also involve the assessment of the upper abdomen, as there are conditions (e.g., gastritis, pancreatitis) which may present with chest pain.

At the moment, ultrasound is used in some emergency departments in the evaluation of chest pain; however, there is not yet a specific scheme to evaluate these patients. Thus, examination is not standardized, and it is not possible to completely understand to what degree it is useful. It is also worth noting that, even though ultrasound presents some strong advantages, it also presents important limitations in the context of the emergency department. In particular, it is time consuming and depends on the experience and competence of the operator. 

However, even though there are limitations, we think that ultrasound, and in particular POCUS, could have an important impact both on physicians and on patients, even though research in this field is still necessary.

## Figures and Tables

**Figure 4:**
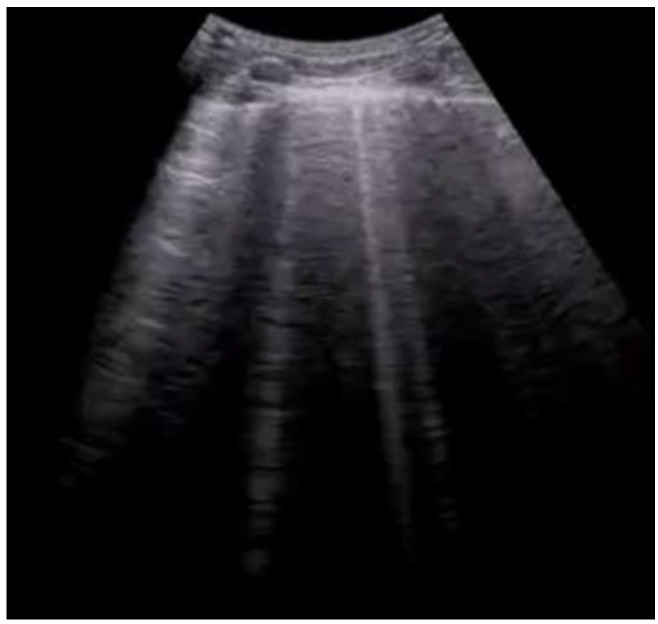
Longitudinal scan with evidence of B-lines: vertical artifacts perpendicular to the pleural line are indicative of inflammation or interstitial edema.

**Figure 5:**
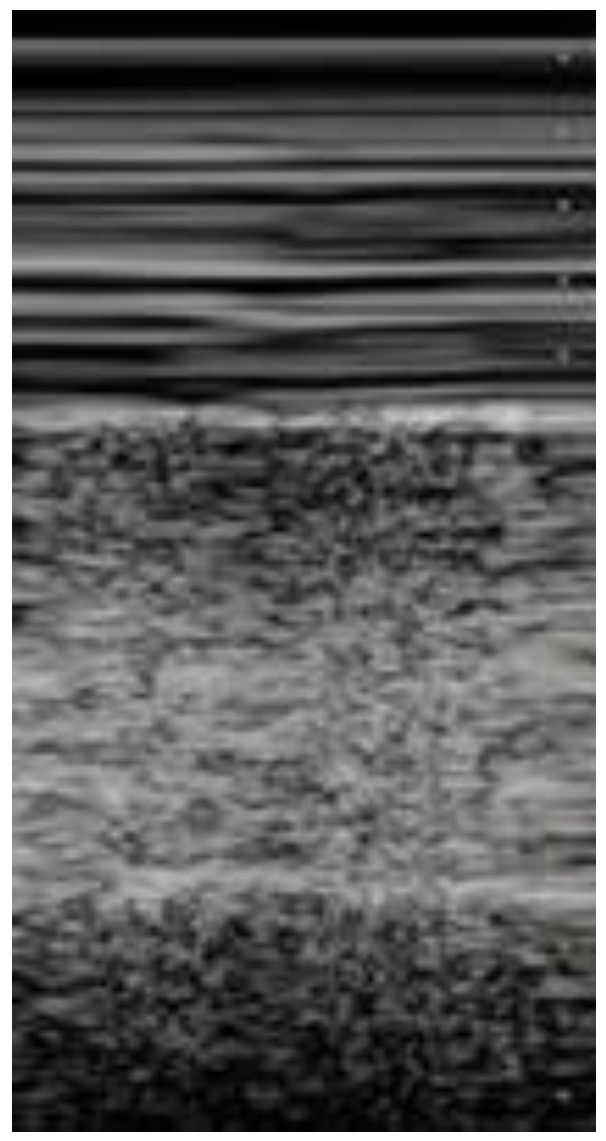
Lung ultrasonography, signs in motion mode (M-mode): seashore sign, indicative of the physiological sliding of the pleural line.

**Figure 6:**
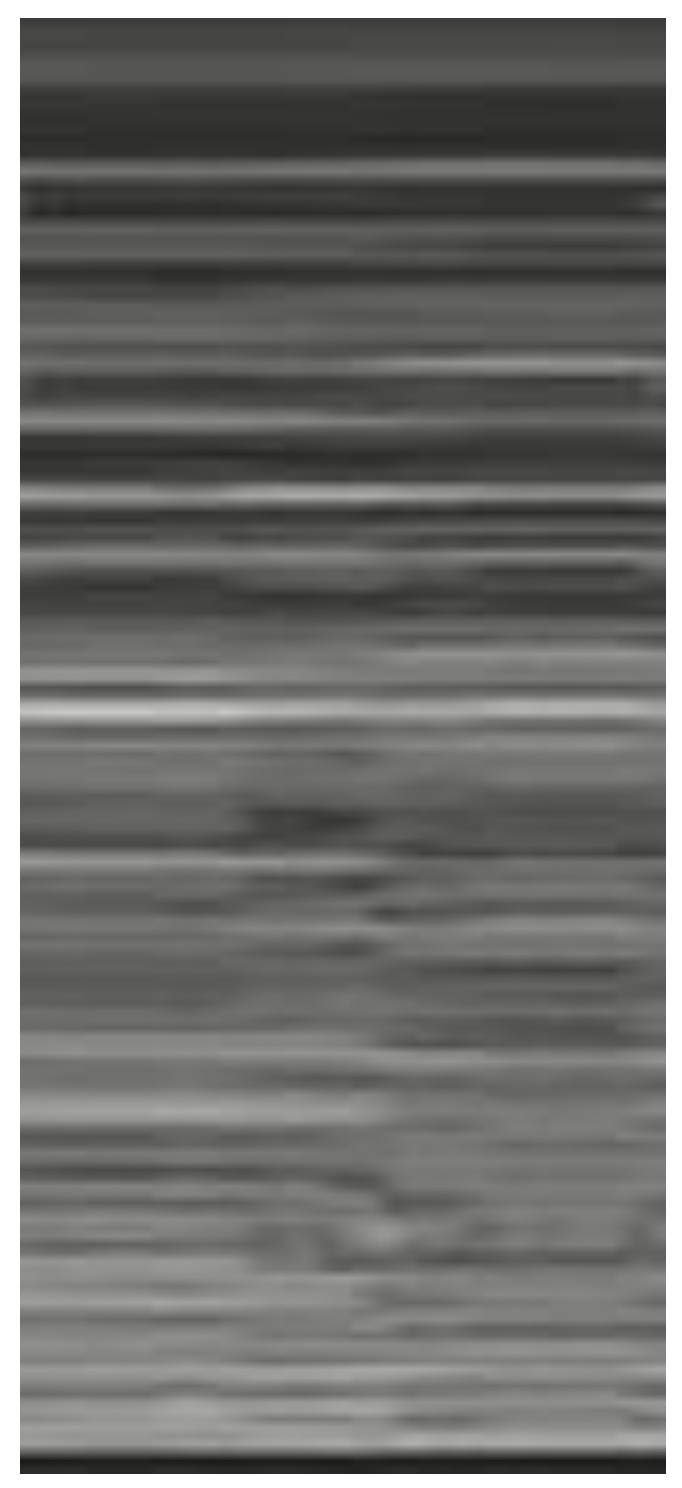
Lung ultrasonography, signs in motion mode (M-mode): barcode sign (sign of the stratosphere), no evidence of pleural sliding, a sign suggestive of pneumothorax.

**Figure 7:**
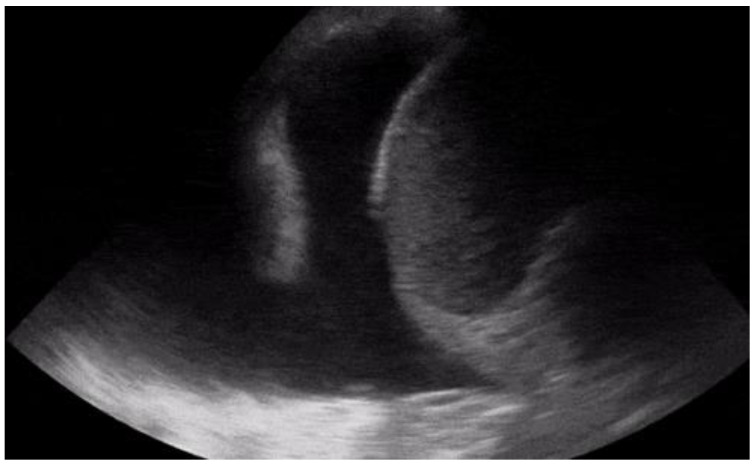
Longitudinal scan with presence of massive pleural effusion above the diaphragmatic line in pleural cavity, with atelectasis of adjacent lung parenchyma.

**Figure 8:**
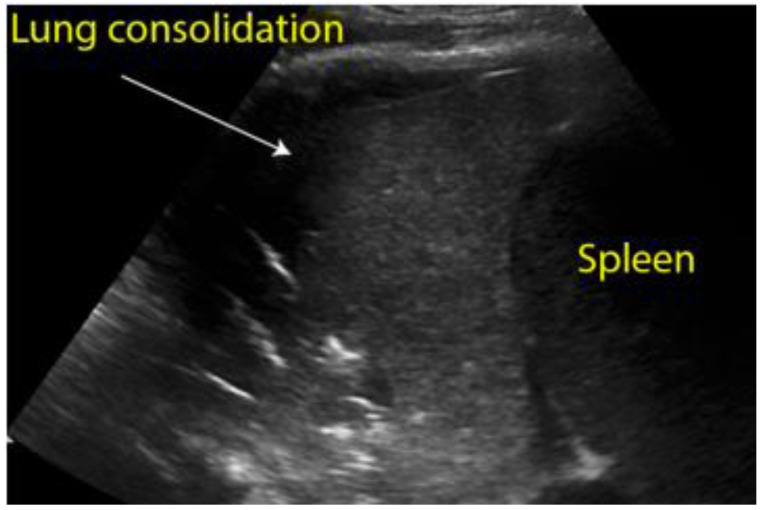
Longitudinal scan of left hypochondrium with presence of lung consolidation suggestive of pneumonia.

**Figure 9:**
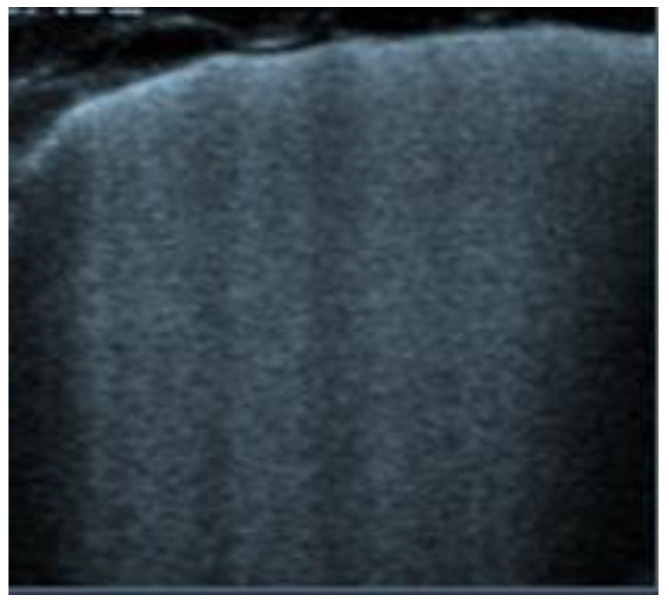
Transverse scan, presence of diffuse B-lines over all fields (white lung) indicative of acute pulmonary edema.

**Table 1:** Relation between inferior vena cava diameter, inspiratory collapse and right atrium pressure.

IVC Diameter	Inspiratory Collapse	Right Atrium Pressure
<2.1 cm	>50%	3 mm Hg (range 0–5 mm Hg)
>2.1 cm	<50%	15 mm Hg (range 10–20 mm Hg)

**Table 2:** POCUS application in cardiac clinical pictures.

Uses of Cardiac POCUS in the Emergency Department	
Disease	Assessment
Pulmonary embolism	Ejection fraction
Heart failure	Inferior vena cava filling
Aortic pathologies	
Pericardial effusion and cardiac tamponade	

**Table 3:** Ultrasound signs in clinical practice.

**Findings**	**Description**	**Interpretation**
**Physiological findings**		
A-Lines	Hyperechoic horizontal lines parallel to the pleural line	Normal findings
Sliding sign	Physiological sliding of the pleural layers during respiratory acts	Normal findings, excluding the presence of PNX
**Pathological findings**		
Liver sign	The lung has the same consistency as the liver	Consolidation
B-Lines (Figure 4)	Vertical artifacts perpendicular to the pleural line	Presence of interstitial infiltrate, suggestive of acute pulmonary edema or COVID-19 pneumonia
**Signs in motion mode (M-mode)**		
Seashore sign (Figure 5)	The pleura is represented by horizontal artifacts and the underlying lung has a sandy pattern.	Normal finding
Barcode sign (sign of the stratosphere) (Figure 6)	Both the pleura and the lung appear as horizontal artifacts	PNX

**Table 4:** Clinical patterns and their ultrasound findings.

Clinical Pattern	Ultrasound Findings
PNX	Absence of B-lines and lung sliding, barcode/stratosphere sign in M-mode
Pleural effusion	Detection of hypoechoic material in the pleural cavity (Figure 7)
Pneumonia	Consolidation, air bronchogram sign (Figure 8)
Pulmonary embolism	Dilation of the right heart cavities or the presence of blood clots within them, and the presence of deep vein thrombosis
Acute pulmonary edema	Presence of diffuse B-lines (Figure 9)

**Table 5:** Applications of thoracic ultrasound.

Applications of Thoracic Ultrasound
Acute pulmonary edema Pneumothorax Hemothorax Pleural effusion Pulmonary embolism COVID-19 Exacerbation of asthma and COPD

**Table 6:** Clinical patterns and their ultrasound findings.

Clinical Pattern	Ultrasound Findings
Gastritis	Thickening of antral walls and mucosal layers
Oesophagitis	Thickening of oesophageal wall; slow, trickling reflux of gastric content
Oesophageal perforation	Non-visualization of the heart on ultrasound, free fluid may also be present in the upper abdominal quadrants
Gastric perforation	Free fluid in the upper abdominal quadrants; hyper echogenicity of the right anterior extrarenal tissue (renal rind sign)
Cholelithiasis	Gallstone with a shadow cone; possible bile duct dilatation.
Cholecystitis	Gallbladder distension, wall oedema, and pericholecystic fluid collection.
Pancreatitis	Enlarged and oedematous pancreas, peripancreatic fluid collections, venous thrombosis, arterial pseudoaneurysm, the presence of gallstones, or dilatation of the biliary tree
Nephrolithiasis	Kidney stone with a shadow cone, dilatation above the stone.

**Table 7:** Diagnostic tools for differential diagnoses.

Patients Who Came to Emergency Department for Chest Pain and Dyspnea	
Diagnostic Suspicion	Role of Diagnostic Tests and POCUS
Acute coronary syndrome (ACS)	Performing ECG and troponin assay
Aortic dissection	The gold standard is represented by CT angiography or trans-oesophageal echocardiography, while POCUS can help in cases where these tests cannot be performed
Pericardial effusion and cardiac tamponade	POCUS is one of the tests that allows diagnosis
Pulmonary embolism	The gold standard is represented by CT angiography, and POCUS can select which patients should undergo this examination
Acute pulmonary edema	POCUS is one of the tests that allows diagnosis
PNX	Chest X-ray is the first-level examination, and thoracic ultrasound is quite accurate. E-FAST is the first choice for the trauma patient.
Pleural effusion or hemothorax	Chest X-ray is the first-level examination, and thoracic ultrasound is quite accurate. E-FAST is the first choice for the trauma patient.
Pneumonia	Blood tests with inflammatory indices and a chest X-ray comprise the first level of examination, and thoracic ultrasound is quite accurate.
COVID-19 pneumonia	The findings of ultrasound changes suggestive of infection can be isolated early; chest CT remains the most accurate examination.
Exacerbation of asthma and COPD	In this case, the finding of a normal type A pattern without pleural or parenchymal changes can be suggestive of these pathologies.

## Data Availability

Not applicable.
